# Single-branched stent-graft with on-table fenestration for endovascular repair of primary retrograde type A aortic dissection: A multicenter retrospective study

**DOI:** 10.3389/fcvm.2022.1034654

**Published:** 2022-11-17

**Authors:** Heng Zhang, Jiaxuan Feng, Hongqiao Zhu, Shun Xiao, Mingyuan Liu, Yili Xu, Dongshan Yang, Junjun Liu, Mingjin Guo

**Affiliations:** ^1^Department of Vascular Surgery, Affiliated Hospital of Qingdao University, Qingdao University, Qingdao, Shandong, China; ^2^Department of Vascular Surgery, Changhai Hospital, Naval Medical University, Shanghai, China; ^3^Department of Vascular Surgery, Beijing Friendship Hospital, Capital Medical University, Beijing, China; ^4^Department of Vascular Surgery, The 960th Hospital of the People’s Liberation Army, Jinan, Shandong, China; ^5^Department of Vascular Surgery, Cangzhou Central Hospital, Cangzhou, Hebei, China

**Keywords:** primary retrograde type A aortic dissection, thrombosis, single-branched stent graft with on-table fenestration, endoleaks, endovascular repair

## Abstract

**Objective:**

This study aims to evaluate the feasibility, efficacy, and safety of a single-branched stent-graft with on-table fenestration for primary retrograde type A aortic dissection (RTAD) during thoracic endovascular aortic repair (TEVAR).

**Materials and methods:**

From January 2019 to December 2021, 36 patients with primary RTAD from five tertiary hospitals received medical management in the acute phase. They underwent TEVAR with a proximal zone 1 landing for aortic arch reconstruction in the subacute phase, using a fenestration technique on a single-branched stent-graft. Nearly 2 weeks after admission, computed tomography angiography (CTA) was re-examined to evaluate the thrombosis status of retrograde false lumen (FL). The primary outcomes were technical success, patency of the target branch arteries, and absence of type Ia endoleaks. The second outcomes were stent-graft-related complications and all-cause mortality.

**Results:**

The mean age was 56.2 ± 11.3 years, and 29 (80.6%) were male. After a median interval of 18.0 [interquartile range (IQR), 17.0–20.3] days of medical treatment, the partial and complete thrombosis of proximal FL rates increased to 52.8% and 47.2%, respectively. One patient (2.8%) experienced postoperative type Ia endoleaks, and was successfully re-treated using coli and Onyx glue. The median hospital stay was 20.5 (IQR, 18.0–23.0) days. The overall technical success rate was 100%. The median follow-up time was 31.5 (IQR, 29.8–34.0) months. There was one death (2.8%) due to gastrointestinal bleeding. Distal aortic segmental enlargement (DASE) occurred in two (5.6%) patients. No major complications or recurrent dissections in the proximal landing zone were recorded during follow up.

**Conclusion:**

The retrograde FL in primary RTAD could realize partial or complete thrombosis after medical management in the acute phase, and it might be regarded as a valid proximal landing zone for endovascular repair. The single-branched stent graft with on-table fenestration performed in the subacute phase may be feasible strategy in selective primary RTAD patients.

## Introduction

Retrograde type A aortic dissection (RTAD) is a special subset of type A aortic dissection (TAAD), whose entry tear is located in the arch or behind, and the retrograde false lumen (FL) involves the ascending aorta (1). RTAD incidence is about 7–25% (2). To treat the entry tear, total arch replacement with the frozen elephant trunk technique was considered a reasonable operation for RTAD patients (3, 4). However, patients with high surgical risks, such as elderly patients or those with preoperative critical organ failure, remain unsuitable for open surgery because of intraoperative hypothermic circulatory arrest (5).

Several studies have reported that thoracic endovascular aortic repair (TEVAR) has emerged as an attractive alternative to open surgery by sealing the entry tear and promoting aortic remodeling (6). However, a “healthy” proximal landing zone (PLZ) was considered the basis for endograft implantation to avoid re-entry caused by the proximal site of the endograft, implying that PLZ for RTAD had to be in the ascending aorta (7). Nevertheless, landing in the ascending aorta was associated with a higher risk of stent-graft-related re-entry tear (8). Therefore, how to land in the aortic arch safely, is an unsettled question for RTAD. In our previous study, retrograde FL thrombosis was induced by using coils and Onyx glue in the stage one procedure, a relatively “healthy” PLZ for stent-graft implantation in the second stage procedure (9). However, only a small number of RTAD patients met the special anatomical feature for coil embolization of retrograde FL.

In this study, we found that primary RTAD tended to develop thrombosis in the proximal FL after nearly 2 weeks of medical management. Drug treatment was usually prescribed to lower aortic wall stress and prevent aortic expansion or rupture in selected patients with aortic dissection (10, 11). On this basis, we evaluated the safety and efficacy of the single-branched stent graft with on-table fenestration during zone 1-landing TEVAR for subacute RTAD.

## Materials and methods

### Study population and procedure

A multicenter, retrospective, cohort study was performed based on the China Collaborator Group for Aortic Dissection (CGAD) (12). There were 579 patients with aortic dissection in five tertiary medical centers from January 2019 to December 2021. Among them, 81 (14.0%) were diagnosed with primary RTAD, which was defined as retrograde FL involving the ascending aorta with an entry tear located in zone 1 or behind (1). In the acute stage, 42 (7.3%) patients who met the inclusion criteria were first enrolled and admitted for medical treatment. During or after nearly 2 weeks of medical management, six (1.0%) patients were excluded due to persistent chest pain or unstable hemodynamics, retrograde FL expansion, and pericardial effusion. Finally, 36 patients (6.2%) were recruited for this cohort study, and they underwent endovascular repair using the novel technique in the subacute stage.

All patients who underwent aortic computed tomography angiography (CTA) were then assessed by a multidisciplinary team, including vascular surgeons, radiologists, and anesthesiologists. The type of aortic arch, location of the primary entry tear, aortic arch pathologies, thrombosis status of retrograde FL, maximum diameter of the aorta, and true or false lumen were assessed using Endosize software (Therenva SAS, Rennes, France). A central vessel reconstruction in central line (CL) protocol reconstruction mode was obtained for all CTAs. The inclusion criteria were as follows: (1) patients diagnosed with primary RTAD; (2) no severe complications, such as mal-perfusion, imminent rupture or rupture, aortic regurgitation, etc.; (3) retrograde FL in partial or complete thrombosis after medical treatment; (4) patients in a relatively stable condition (Supplementary Figure 1). This cohort study was reported by strengthening the reporting of observational studies in epidemiology (STROBE) statement for cohort studies. This study was approved by the Institutional Review Board and the Research Ethics Committee of each center (QYFY WZLL 27010).

### Pre-operative medical management and evaluation

**FIGURE 1:**
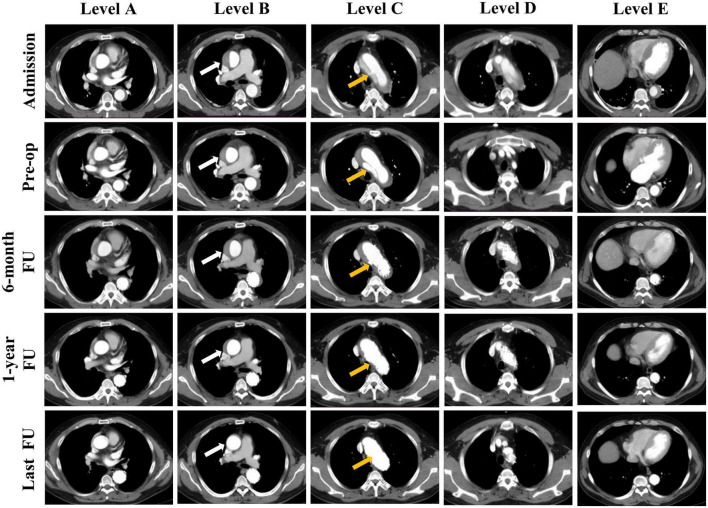
Representative cases of the status of the false lumen thrombosis in different levels of the aorta. Computed tomography angiography (CTA) images show the false lumen (FL) status of a patient at admission, before operation and during follow up. Results of five levels CTA images at different time points are presented: Sinotubular junction (Level A), mid ascending aorta (Level B), proximal aortic arch (Level C), mid aortic arch (Level D), and mid descending aorta (Level E). CTA images show the FL status of the mid ascending aorta at different time points: patent, partial thrombosis, complete thrombosis, partial absorption, completely absorption (Level B, white arrow). And the entry tear was completely obliterated by stent graft (Level C, yellow arrow).

Strict blood pressure and heart rate control medicines were administered by infusion and/or oral administration to meet the targets for all patients (SBP < 120 mmHg and heart rate < 70 beat/min) (13). The three main anti-hypertension drugs were beta-blockers, calcium channel blockers (CCB), and angiotensin-converting enzyme inhibitors (ACEIs). The thrombosis status of the retrograde FL in the ascending aorta and aortic arch was assessed using delayed phase imaging (Figure 1) (14).

Acute aortic dissection was defined as aortic dissection within 14 days of symptom onset and the subacute in 15–90 days (1). During medical management, the indications of an emergency intervention included persistent chest pain, hemodynamic instability, the proximal FL significant expansion or peri-aorta or pericardial effusion (11).

### Modified fenestrated stent-graft and customization

**FIGURE 2:**
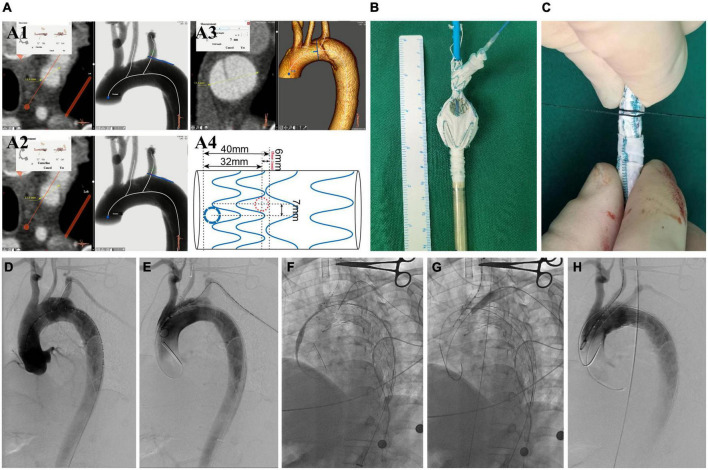
The surgeon’s details modified fenestrated stent graft and the intraoperative images of performing this endovascular technique. **(A1)** Measuring the diameter of LCCA and the offset angle between LCCA and LSA. **(A2)** The diameter of LSA. **(A3)** The distance between the tear entry and the proximal side of the stent. **(A4)** A design for preparing the modified fenestrated stent graft. **(B)** The unsheathed custom-made fenestrated single-branched Castor stent graft and the distal circular fenestration site are selected for LSA. **(C)** Photography of stent-graft when re-sheathed. **(D)** DSA images in the left anterior oblique view. **(E)** Delivery and advancement of the stent graft into the aortic arch. **(F)** The hydrophilic guidewire is passed cephalad into the sheath through a needle hole and advanced through the stent-graft lumen, exiting through LSA fenestration and continuing cephalad external to the stent-graft. **(G)** On-table covered stent was implanted and balloon dilatation. **(H)** DSA shows the patency of aortic arch branches and no endoleak. LCCA, left common carotid artery; LSA, left subclavian artery; CTA, computed tomography angiography; DSA, digital subtraction angiography.

The single-branched Castor stent graft (MicroPort, Shanghai, China) was constructed of woven polyester fabric sewn to self-expanding Nitinol stents, without a proximal or distal bare stent (15). The branch section of the Castor stent graft was intended for the left common carotid artery (LCCA), and surgeons made an on-table fenestration to preserve the left subclavian artery (LSA). Every section of the major stent graft was constrained by string separately, and the strings for all sections could be released by pulling the trigger wire. Consequently, this allowed surgeons to cut on-table or two sting rings to partially release the relevant sections of the major stent graft to make a fenestration in it. The delivery system consisted of an outer and an inner soft sheath. After the fenestration, the major stent graft was re-sheathed in an inner soft sheath followed by an outer sheath (Figures 2A–C).

### Endovascular procedure

All procedures were performed under general anesthesia in a hybrid DSA room. PLZ was planned in zone 1 according to the Ishimaru classification (16). The oversize of the stent graft was less than 5%, according to the maximum diameter of true lumen (TL) in zone 1 on preoperative CTA (17). The proximal sealing length is the distance between the proximal side of the endograft and the entry tear (11). The detailed manipulation of the Castor stent-graft was described earlier (15). The manipulation differences in this study were that the branch section was put into LCCA, and LSA was reconstructed by the on-table customized fenestration on the stent graft, followed by the implantation of a bridge-covered stent graft. The complete exclusion of the entry tear and patency of the super-arch arteries were confirmed in the procedure (Figures 2D–H).

### Follow-up outcomes and aortic remodeling assessments

**FIGURE 3:**
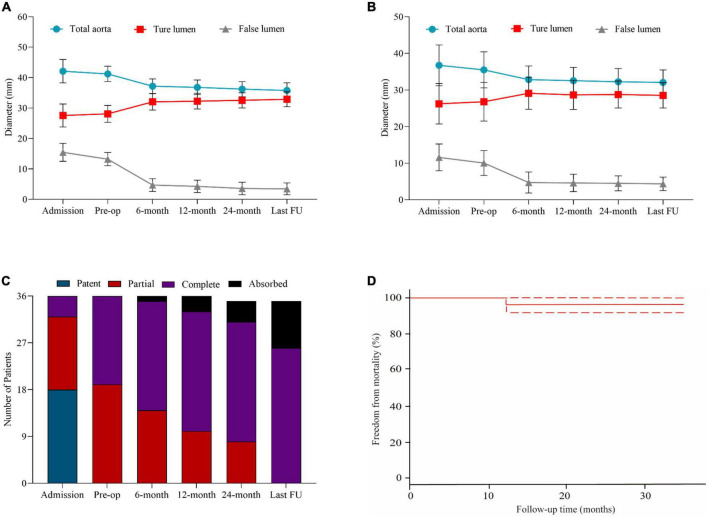
Statistical analysis of aortic remodeling and survival probability during follow-up. **(A)** The changes of diameters of the total aorta, true lumen, and false lumen in the ascending aorta in patients at admission, before the operation, at 6, 12, 24 months, and the latest follow-up. **(B)** The changes of diameters of the total aorta, TL, and FL in the descending aorta in patients at admission, before the operation, at 6, 12, 24 months, and the latest follow-up. **(C)** The status of thrombosed proximal FL in patients in the ascending aorta at admission, before the operation, at 6, 12, 24 months, and the latest follow-up. **(D)** The probability of free from morality during follow-up every 3 months in all patients.

Aortic CTA was performed at six-month intervals for the first year after the intervention, and then annually (Figure 1). The patients had drug treatment during follow-up period. Overall aorta, TL, and FL diameters were measured at the mid ascending and mid descending aorta levels (18) (Figures 3A,B). Three-dimensional vascular images reconstructed from CTA in 1 year and at the end of follow-up were used to evaluate the thrombosis status of FL, the patency of branch arteries, and especially the changes of the retrograde FL. The thrombosis status of FL was assessed using delayed phase imaging (Figure 3C). Clinical follow-up data were obtained from an outpatient clinic or *via* telephone-table interviews. An additional CTA examination or cerebral MRI was conducted if the patients manifested any new adverse symptoms.

### Statistical analysis

Categorical variables were presented as frequencies or percentages. Continuous variables were recorded as mean ± standard deviation (SD) or median [interquartile range (IQR)] and compared using the *t*-test or Wilcoxon rank-sum test, as appropriate. The overall survival was presented using Kaplan–Meier curve method. All statistical analyses were conducted with SPSS version 20.0 (SPSS, Inc., Chicago, IL USA) and graphs were drawn using GraphPad Prism 8.0.1 (GraphPad, Inc., California, USA). A *p*-value < 0.05 was considered significant.

## Results

### Baseline clinical characteristics

Table 1 shows detailed information about the baseline characteristics. The mean age was 56.2 ± 11.3 years. There were 29 male (80.6%) and seven female (19.4%) patients. The top five comorbidities were hypertension (*n* = 31, 86.1%), smoking history (*n* = 17, 47.2%), diabetes mellitus (*n* = 4, 11.1%), history of coronary artery disease (*n* = 3, 8.3%) and prior transient ischemic attack (TIA)/stroke (*n* = 3, 8.3%). There were 29 (80.6%) cases of type I arch and seven (19.4%) of type II arch (19). The primary entry tears of aortic dissection originated in zone 2 (*n* = 14, 38.9%) and zone 3 (*n* = 22, 61.1%). The median interval from admission onset to endovascular procedure was 18.0 (IQR, 17.0–20.3) days.

**TABLE 1:** Baseline characteristics and pre-operative medical management.

Variables	Patients (*n* = 36)
**Demography data**	
Age, years	56.2 ± 11.3
Male	29 (80.6)
**Comorbidities**	
Hypertension	31 (86.1)
Smoking history	17 (47.2)
Diabetes mellitus	4 (11.1)
Coronary artery disease	3 (8.3)
Prior TIA/stroke	3 (8.3)
Chronic obstructive pulmonary disease	1 (2.8)
Renal insufficiency	2 (5.6)
Connective tissue disease	0 (0.0)
Peripheral artery disease	2 (5.6)
Previous aortic surgery	0 (0.0)
**Aortic type arch**	
Type I	29 (80.6)
Type II	7 (19.4)
**Location of primary entry tear**	
Zone 1	0 (0.0)
Zone 2	14 (38.9)
Zone 3	22 (61.1)
**Proximal extent of the dissection pathology**	
Zone 0	36 (100.0)
**Phase of dissection on admission**	
Hyperacute, <1 day	25 (69.4)
Acute, 1–14 days	11 (30.6)
**Medical management**	
Beta-blocker	32 (88.9)
CCB	29 (80.6)
ACEI	14 (38.9)
ARB	13 (36.1)
Interval from medical management to operation, days	18.0 (17.0–20.3)

Categorical variables are presented as *n* (%), and continuous variables are presented as mean ± standard deviation as appropriate. TIA, transient ischemic attack; CCB, calcium channel blocker; ACEI, angiotensin-converting enzyme inhibitor; ARB, angiotensin receptor blocker.

### Pre-operative evaluation

Table 2 summarizes the pre-operative evaluation. The mean admissions and preoperative heart rates were 91.5 ± 8.3 and 67.2 ± 3.7 beats/min, respectively (*p* < 0.001). The mean admissions and preoperative systolic blood pressure (SBP) were 164.1 ± 6.2 and 125.1 ± 3.5 mmHg, respectively (*p* < 0.001). The mean admissions and preoperative diastolic blood pressure (DBP) were 80.2 ± 13.0 and 72.1 ± 5.8 mmHg, respectively (*p* = 0.003). At admission, partial and complete thrombosis occurred in 14 patients (38.9%) and four (11.1%) in the retrograde FL of the ascending aorta. Partial and complete thrombosis of retrograde FL in the aortic arch was found in 19 (52.8%) and 17 (47.2%) patients before TEVAR (Figure 1). The maximal diameter of FL in the ascending aorta decreased after medical treatment (*p* < 0.001).

**TABLE 2:** Preoperative evaluation of primary retrograde type A aortic dissection in 36 patients.

Variables	Admission	Preoperative	*P-value*
Heart rate, beat/min	91.5 ± 8.3	67.2 ± 3.7	<0.001
**Blood pressure, mmHg**			
SBP	164.1 ± 6.2	125.1 ± 3.5	<0.001
DBP	80.2 ± 13.0	72.1 ± 5.8	0.003
**CTA features**			
**Thrombosis of retrograde FL**			
Patent	18 (50.0)	0 (0.0)	<0.001
Partial thrombosis	14 (38.9)	19 (52.8)	0.34
Complete thrombosis	4 (11.1)	17 (47.2)	<0.001
Maximal diameter of the total aorta in ascending aorta, mm	42.1 ± 3.8	41.2 ± 2.3	0.28
Maximal diameter of FL in ascending aorta, mm	15.5 ± 2.9	13.2 ± 2.9	<0.001
Maximal diameter of TL in ascending aorta, mm	27.6 ± 3.8	29.5 ± 2.8	0.03
Maximal diameter of descending aorta, mm	36.7 ± 2.3	35.5 ± 2.4	0.05

Categorical variables are presented as *n* (%), and continuous variables are presented as mean ± standard deviation as appropriate. SBP, systolic blood pressure; DBP, diastolic blood pressure; CTA, computer tomography angiography; FL, false lumen; TL, true lumen.

### Details of the procedure

The operative details and outcomes are listed in Table 3. The single-branched stent-grafts were deployed at the planned position, whose PLZ was in zone 1. The mean duration of the procedure was 105.1 ± 13.8 min. The proximal and distal median diameters of the major stent graft were 36.0 (IQR, 28.0–38.0) and 28.0 (IQR, 22.0–30.0) mm, respectively. The mean length of PLZ was 45.6 ± 7.4 mm. The mean branch length for LCCA was 29.9 ± 1.2 mm. The mean diameter and length of the bridge endograft for LSA were 8.6 ± 1.5 and 45.8 ± 5.5 mm, respectively. The bridge endograft for LSA included Viabahn (W.L. Gore & Associates, USA; *n* = 21) and Lifestream (BD, USA; *n* = 15). The final angiography confirmed complete exclusion of the entry tear in 36 cases, without type I/III endoleaks (Figure 2H).

**TABLE 3:** Operative procedures and outcomes of 36 patients treated by thoracic endovascular repair (TEVAR) for primary retrograde type A aortic dissection.

Variables	Patients (*n* = 36)
Operation time, min	105.1 ± 13.8
**Proximal landing zone**	
Zone 1	36 (100.0)
Median diameter, mm	36.0 (28.0–38.0)
**Distal landing zone**	
Median diameter, mm	28.0 (22.0–30.0)
Length of the proximal landing zone, mm	45.6 ± 7.4
**Reconstruction of the LCCA (branch section)**	
Diameter, mm	10.6 ± 1.8
Length, mm	29.9 ± 1.2
Brand	–
Castor stent graft (MicroPort, Shanghai, China)	36 (100.0)
**Reconstruction of the LSA**	
Diameter, mm	8.6 ± 1.5
Length, mm	45.8 ± 5.5
Brand	–
Viabahn (W.L. Gore & Associates, USA)	21 (58.3)
Lifestream (BD, USA)	15 (41.7)
**Patient outcomes**	
Median hospital stays, days	20.5 (18.0–23.0)
Median follow-up time, days	31.5 (29.8–34.0)
30 days mortality	0 (0.0)
30 days morbidity	1 (2.8)
Morbidity types	–
Type Ia endoleak	1 (2.8)
Type Ib endoleak	0 (0.0)
Cerebral vascular event	0 (0.0)
Spinal cord infarction	0 (0.0)
Branch artery occlusion	0 (0.0)
RTAD	0 (0.0)
SINE	0 (0.0)
DASE	2 (5.6)
Reintervention	0 (0.0)

Data are presented as *n* (%) or median (interquartile range) or mean ± standard deviation as appropriate. LCCA, left common carotid artery; LSA, left subclavian artery; RTAD, retrograde type A aortic dissection; SINE, stent graft induced new entry tear; DASE, distal aortic segmental enlargement.

### Post-operative and follow-up outcome

Detailed information about the follow-up outcomes is presented in Table 3. The median hospital stay was 20.5 (IQR, 18.0–23.0) days. One (2.8%) patient with type Ia endoleak was observed within 30 days post-operation, and was successfully re-treated using coli and Onyx glue. No other adverse events were found in the early outcomes. The median follow-up time was 31.5 (range: 29.8–34.0) months. Two (5.6%) patients showed DASE. The follow-up recorded no cerebral vascular event, spinal cord infarction, branch stent occlusion, RTAD, or stent-graft-induced new entry tear (SINE).

### Morphological analysis in aortic remodeling

The patients’ preoperative and post-operative aortic remodeling are presented in Table 4. The mean diameters of the ascending aorta were 41.2 ± 2.3 and 35.8 ± 3.0 mm in the preoperative and post-TEVAR periods, respectively (*p* < 0.001). The diameter of FL in the ascending and descending aorta shrank by 9.8 ± 2.3 and 4.9 ± 2.4 mm, respectively. The status of retrograde FL in the aortic arch was observed in 35 (97.2%) patients, including complete thrombosis in 26 (72.2%) and total absorption in nine (25.0%) patients (Figure 3C). Figure 3D displays the freedom from mortality of 36 patients.

**TABLE 4:** Aortic diameters of the 36 patients before and after thoracic endovascular aortic repair (TEVAR) for primary retrograde type A aortic dissection.

Diameters	Pre-TEVAR (*n* = 36)	Post-TEVAR (*n* = 36)	Diameter difference	*P-value*
**Ascending aorta**				
Aortic diameter	41.2 ± 2.3	35.8 ± 3.0	–5.4 ± 1.6	<0.001
FL diameter or thickness	13.2 ± 2.9	3.4 ± 1.6	–9.8 ± 2.3	<0.001
**Descending aorta**				
Aortic diameter	35.5 ± 2.4	31.6 ± 3.5	–3.9 ± 2.1	<0.001
FL diameter or thickness	10.1 ± 2.9	4.3 ± 1.8	–4.9 ± 2.4	<0.001

Data are presented as mean ± standard deviation. TEVAR, thoracic endovascular aortic repair; FL, false lumen.

## Discussion

Since described first by Nienaber and Dake in 1999, TEVAR has evolved as a selective treatment strategy for aortic diseases, especially for type B dissection (20). Following the reporting standards of the Society for Vascular Surgery (SVS)/Society of Thoracic Surgeons (STS), retrograde dissection into the ascending aorta with an entry tear located in zone 1 or beyond is classified as type B aortic dissections (TB_0_AD) (1). The subscript “0” of TB_0_AD describes the proximal zone of the aorta involved. A recent study indicated that RTAD had special anatomical features, which may have more stable hemodynamics, slower progress of dissection, and even better prognosis than typical TAAD although the ascending aorta was affected (21). However, the endovascular repair for aortic dissection involving the ascending aorta is more challenging, because it requires the endograft to go more proximally in the ascending aorta to land in a healthy aorta. Therefore, the endovascular management of RTAD is far from consensus.

Previous studies have reported that fenestration and chimney techniques may be feasible for treating some aortic arch pathologies while preserving the blood supply of the brain and upper extremities in selected patients (22). However, the incidence of intraoperative endoleaks and the risk of cerebral infarction were underestimated for the chimney technique (23), especially in cases with an entry tear located very close to the beginning of branch arteries. When the fenestrated devices are used in the aortic arch, aligning the fenestration with the orifice of the branch arteries is difficult and risky due to the curve of the aortic arch and the circumferential angles of supra-arch arteries.

The timing of TEVAR for TBAD is significantly associated with in-hospital/30 days mortality and stroke (24). In a previous study with 31 RTAD cases treated with TEVAR, 24 (77.4%) patients received the early intervention (<14 days after onset), but the rate of stent-graft-related complications at 1 year was high as 42% (25). Several studies have also shown that TEVAR for TBAD delayed at least 15 days from symptom onset helps improve the long-term survival rate (24). Furthermore, it has been reported that the subacute phase may be potentially the better timing of treatment to perform TEVAR (26). In this study, RTAD patients received TEVAR in the subacute phase, using a fenestration technique on a single-branched stent-graft for aortic arch reconstruction.

Kato et al. indicated that the implantation on complete FL thrombosis is safe, and TEVAR was carefully applied in RTAD patients, with no perioperative deaths (27). This is an important reason that the proximal FL thrombosis is a marker of reduced FL pressure (14). In our previous study, the thrombosed proximal FL induced by coils and glue might serve as a relatively “healthy” PLZ for endovascular repair, showing positive aortic remodeling in the follow-up period (9). However, Urbanski et al. implied that the pigtail catheter for angiography of FL could induce retrograde progression of the dissection and weaken the proximal aspect of the aortic wall (28). Meanwhile, this “two-stages procedure” might increase cerebral infarction risk because of excessive aortic arch operations (29). Neither RTAD nor late aortic-related death was observed due to the oversize of the stent graft no more than 5% at PLZ, and no proximal or distal bare stent. And the reason why the type Ia endoleak occurred in one patient may be partial thrombosis in the retrograde FL. It was difficult to determine the proper proximal size of stent-graft because the partial thrombosis of the retrograde false lumen left uncertainty of aortic arch.

To reduce the cerebral malperfusion, rapid supra-arch artery revascularization is demanding. This situation has developed *in situ* fenestration using needles, lasers, or radiofrequency punctures (30). However, the above technique takes time to penetrate the main stent grafts, which may increase the risk of cerebral infarction. In this study, the design of the stent graft was inspired by the construction of a single-branched Castor stent graft, which has been confirmed safe and effective for aortic arch pathology (15). The anchored and referenced effects of the branch section can help this procedure in moving the designed location quickly and accurately for rapid revascularization in this study.

The study has several limitations. It is a retrospective study without a control group. First, the study lacks comparison between single-branched stent-graft with on-table fenestration, and chimney technique. Second, the study is small and highly selected patients with the retrograde false lumen of partial or complete thrombosis.

## Conclusion

The thrombosed retrograde FL in the aortic arch of primary RTAD could be expected after strict medical management, which might be regarded as a valid PLZ for endovascular repair. The single-branched stent-graft with on-table fenestration performed in the subacute phase may be a feasible strategy for in selective primary RTAD patients. Further studies with larger samples size and longer follow-up period at more centers is required.

## Data availability statement

The original contributions presented in this study are included in the article/Supplementary material, further inquiries can be directed to the corresponding authors.

## Ethics statement

This retrospective study involving participants were reviewed and approved by the Affiliated Hospital of Qingdao University, Changhai Hospital, Naval Medical University, Beijing Friendship Hospital, Capital Medical University, The 960th Hospital of the People’s Liberation Army, Cangzhou Central Hospital.

## Author contributions

MG and JL conceptualized and led the work. HZ, JF, and HZ were contributed to design the study and wrote the article. SX, ML, YX, and DY were contributed to the data collection, analysis, and interpretation. HZ and JF contributed to the writing, review, editing, and supervision. All authors have read and approved the final version of the manuscript.

## Supplementary material

Supplementary Figure 1 Consort diagram for surgical treatment of primary retrograde type A aortic dissection (2019–2021, *n* = 579). RTAD, retrograde type A aortic dissection; FL, false lumen; TEVAR, thoracic endovascular aortic repair.
