# Supplementary figures and images for: Single-branched stent-graft with on-table fenestration for endovascular repair of primary retrograde type A aortic dissection: A multicenter retrospective study
Source: Front Cardiovasc Med. 2022 Nov 17;9:1034654. doi: 10.3389/fcvm.2022.1034654 (PMC9713701; doi:10.3389/fcvm.2022.1034654)

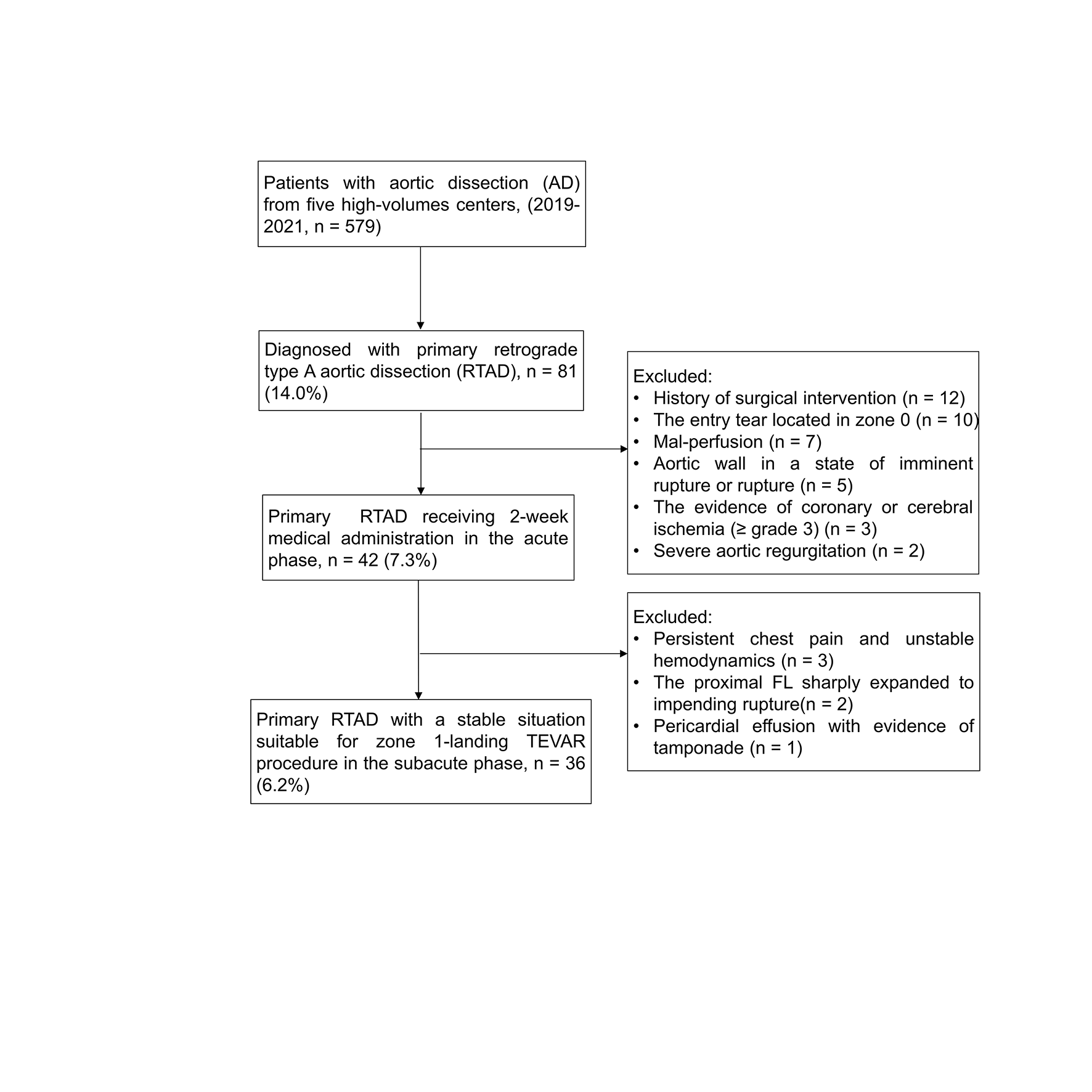

Supplement: Supplementary Figure 1 — Consort diagram for surgical treatment of primary retrograde type A aortic dissection (2019–2021, n = 579). RTAD, retrograde type A aortic dissection; FL, false lumen; TEVAR, thoracic endovascular aortic repair. [file Image_1.TIF]

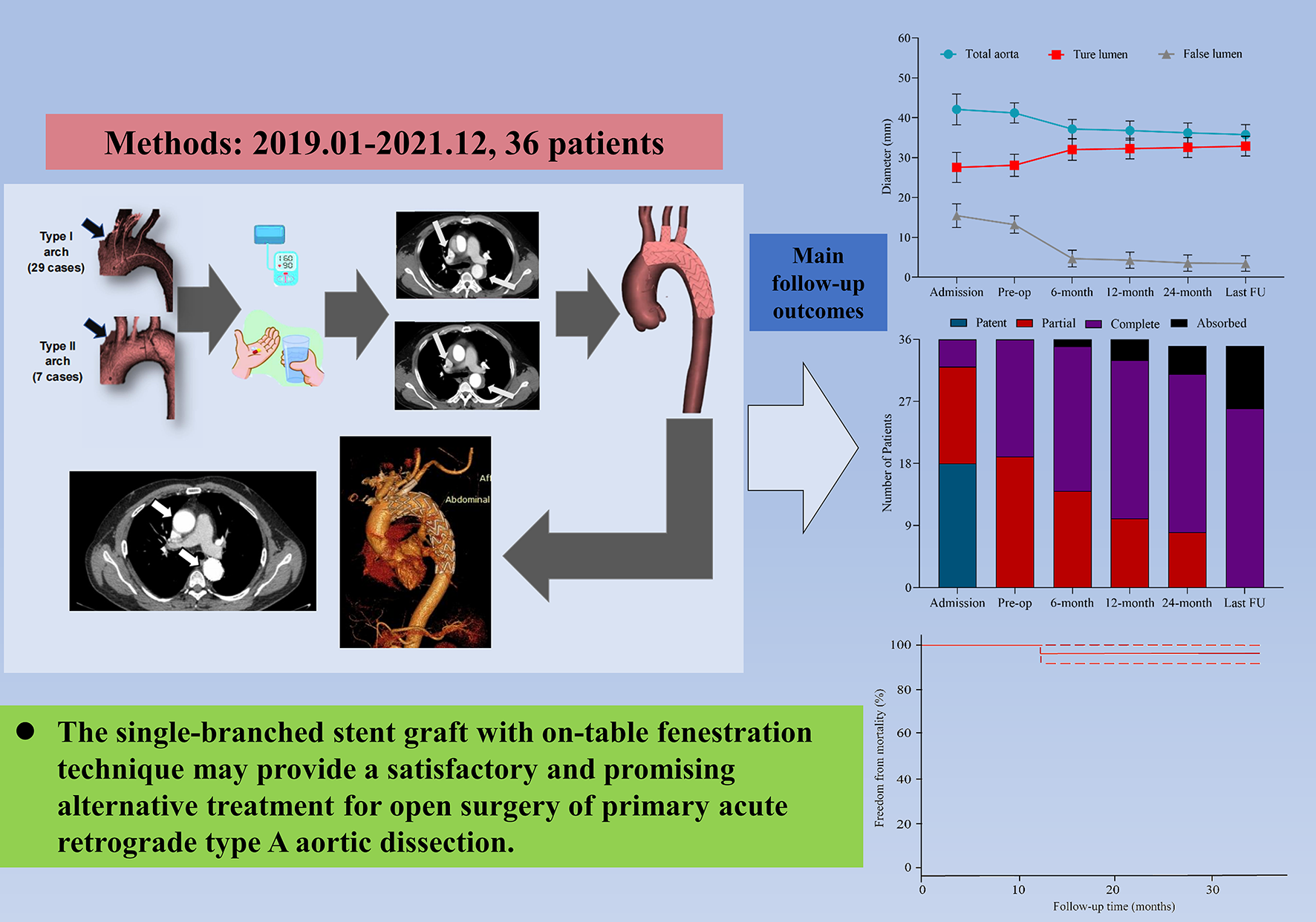

Supplement: Supplementary file 2 [file Image_2.TIF]
